# Light intensity effects on bioproduct recovery from fuel synthesis wastewater using purple phototrophic bacteria in a hybrid biofilm-suspended growth system

**DOI:** 10.1016/j.btre.2024.e00863

**Published:** 2024-11-06

**Authors:** Sultan Shaikh, Gordon McKay, Hamish Robert Mackey

**Affiliations:** a Division of Sustainable Development, College of Science and Engineering, Hamad bin Khalifa University, Qatar Foundation, Doha, Qatar; b Department of Civil and Natural Resources Engineering, University of Canterbury, Private Bag 4800, Christchurch 8140, New Zealand

**Keywords:** Fuel-synthesis wastewater, Purple non-sulphur bacteria, Light intensity, Biofilm, Bioproduct recovery

## Abstract

This research looked at how three different light intensities (1600, 4300, and 7200 lx) affect the biomass development, treatment of fuel synthesis wastewater and the recovery of valuable bioproducts between biofilm and suspended growth in a purple-bacteria enriched photobioreactor. Each condition was run in duplicate using an agricultural shade cloth as the biofilm support media in a continuously mixed batch reactor. The results showed that the highest chemical oxygen demand (COD) removal rate (56.8 ± 0.9 %) was found under the highest light intensity (7200 lx), which also led to the most biofilm formation and highest biofilm biomass production (1225 ± 95.7 mg). The maximum carotenoids (Crts) and bacteriochlorophylls (BChls) content occurred in the suspended growth of the 7200 lx reactor. BChls decreased with light intensity in suspended growth, while in biofilm both Crts and BChls were relatively stable between light conditions, likely due to an averaging effect as biofilm thickened at higher light intensity. Light intensity did not affect protein content of the biomass, however, biofilm showed a lower average (41.2 % to 43.7 %) than suspended biomass (45.4 % to 47.7 %). For polyhydroxybutyrate (PHB) the highest cell concentration in biofilm occurred at 1600 lx (11.4 ± 2.4 %), while for suspended growth it occurred at 7200 lx (22.7 ± 0.3 %), though total PHB productivity remained similar between reactors. Shading effects from the externally located biofilm could explain most variations in bioproduct distribution. Overall, these findings suggest that controlling light intensity can effectively influence the treatment of fuel synthesis wastewater and the recovery of valuable bioproducts in a biofilm photobioreactor.

## Highlights

• Effect of three light intensities (1600/4300/7200 lx) was examined.
• The highest intensity (7200 lx) showed maximum COD removal and biofilm formation.
• Optimal intensity for PHB differed for biofilm (low) and suspended growth (high).
• Light intensity did not affect cellular protein content.
• Complex behavior, particularly with pigments, occurs due to shading from biofilm.

## Graphical abstract

**Figure:**
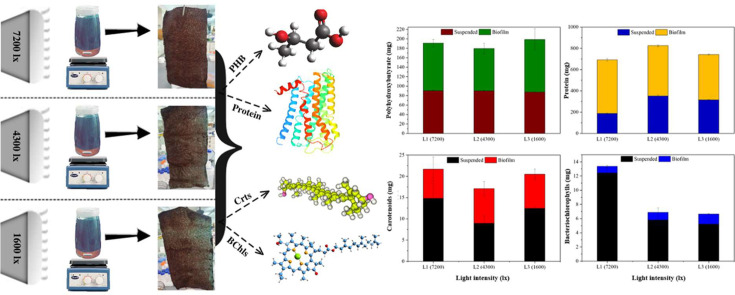

## 1 Introduction

As the demand for cleaner and more sustainable fuels continues to grow, the production of synthetic fuels is expected to grow at a compounding annual growth rate of around 5.4 % (“Synthetic Fuels Market, Industry Share Forecast Report, [1],” n.d.). This expected growth would also correspond to a growth in the generation of fuel synthesis wastewater (FSW). FSW is a byproduct from the production of liquid fuel, which may come from a number of different processes. One of the major routes is from natural gas using the Fischer–Tropsch process, with the largest facility alone producing 45,000 m^3^/day of FSW [2]. Fischer–Tropsch FSW is a highly acidic and organic-rich liquid, with a high chemical oxygen demand (COD) of up to 32 g/L, containing primarily dissolved organic acids and alcohols [3]. The traditional methods for the treatment of FSW are often energy-intensive and generate significant quantities of secondary waste.

Purple non-sulfur bacteria (PNSB) offer a potentially sustainable and cost-effective solution for the treatment of FSW. PNSB can convert organic compounds in FSW into various bioresources. This approach may also have cost-offsetting benefits by reducing the amount of wastewater that requires conventional treatment and disposal. PNSB are a diverse group of phototrophic bacteria that can degrade a range of organic pollutants in FSW and produce valuable bioproducts such as carotenoids (Crts), bacteriochlorophylls (BChls), single cell protein (SCP), and polyhydroxybutyrate (PHB) [4]. The production of these bioproducts from FSW conserves natural resources and reduces waste, while also offering economic benefits through new market creation and reduced waste management costs. Therefore, the use of PNSB for FSW treatment represents a promising approach that offers multiple benefits, both in terms of environmental sustainability and economic viability.

Biofilm photobioreactors are receiving increased interest for the treatment of wastewater and resource recovery [[5], [6], [7], [8]], as they provide a number of potential advantages over traditional photobioreactors. The high biomass density of biofilm provides several benefits. Firstly, it enables a more energy-efficient operation and harvesting because high biomass concentration requires minimal dewatering before downstream processing. Additionally, the accumulation of biomass in the biofilm leads to a significant reduction in reactor size and medium volume needed to cultivate the same amount of biomass [9]. This makes biofilms a suitable option for the treatment of FSW as well as for resource recovery [10].

Light intensity is a critical factor that affects the treatment process, as it plays a crucial role in photosynthesis and determines the rate of growth, substrate degradation and therefore efficiency of the process [11]. Previous studies have investigated the effect of light intensity on wastewater treatment [[12], [13], [14]], but these studies focused on different types of wastewaters and most did not consider light effects on biofilm formation. However, these studies provide important insights into the role of light intensity on PNSB. Previous research has shown that while an optimal light intensity can promote the production of valuable bioproducts, too much or too little light can negatively impact microbial performance [[15], [16], [17]]. It is important to note that the optimal light intensity for a particular mixed microbial culture may vary depending on the proportion of phototrophic cells and different species present in the culture. As such, understanding the interplay between light intensity and mixed microbial culture is critical for the development of effective wastewater treatment strategies [11].

Despite the lack of specific studies on FSW treatment and biofilm formation, the findings of these previous studies highlight the importance of investigating the impact of light intensity on these processes. The present research aims to investigate the impact of light intensity at three levels (7200, 4300, and 1600 lx) on FSW treatment and the recovery of valuable bioproducts in a PNSB enriched biofilm photobioreactor. In particular, the study will assess the relative growth and bioproduct production in the suspended biomass and biofilm fractions. By examining the impact of light intensity on FSW treatment and biofilm formation, it is possible to gain a better understanding of the optimal conditions for the treatment process and to identify strategies for maximizing the production of valuable bioproducts. This study aligns with the principles of a circular economy by closing the loop on resource use and waste management, promoting sustainable and efficient resource utilization. The research questions of this study are (a) What is the impact of light intensity on the treatment of FSW by PNSB; (b) What role does light intensity play in the formation of PNSB biofilm; (c) How does light intensity influence the recovery of value-added bioproducts such as Crts, BChls, protein, and PHB; and (d) How does interaction between biofilm and suspended growth in such a system, based on available light penetration, affect value-added bioproducts in these two modes of growth.

## 2 Materials and methods

### 2.1 Biofilm photobioreactor and growth conditions

The study utilized three laboratory-scale glass photobioreactors, each with a 2 L reactor volume and 1.7 L working volume. Experiments were conducted under room temperature conditions (25–30 °C), and the photobioreactors were exposed to three different light intensities: 7200 lx (56 W/m^2^) (L1), 4300 lx (34 W/m^2^) (L2), and 1600 lx (13 W/m^2^) (L3) using a 30 W flood white light. The light intensity was adjusted by varying the distance between the wall of the photobioreactor and light source. An agricultural shade cloth, measuring 30.48 cm x 15.24 cm and pre-washed with distilled water, was used as the biofilm support material. The mesh size of the agricultural shade cloth had openings of approximately 600–800 µm. The shade cloth was selected due to its ability to provide a suitable environment for biofilm growth, offering both light passage and physical support. Previous research had also demonstrated its potential for PNSB biofilm formation [4,18].

**Fig. 1:**
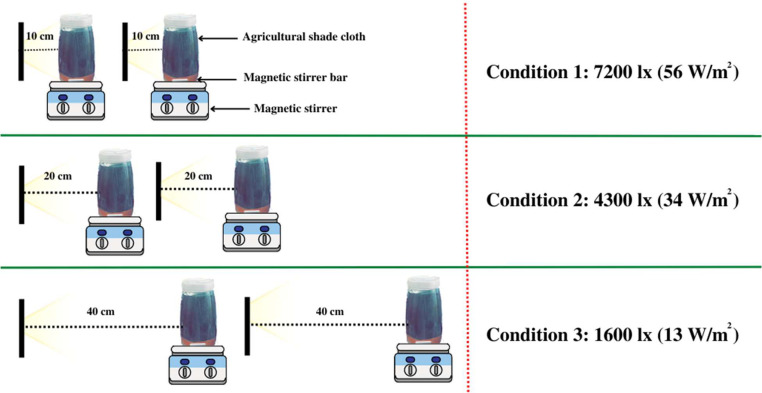
Experimental setup used in the study.

All experiments were run in biological duplicates and were performed under illluminated-anaerobic conditions. The photobioreactors were agitated at 200 rpm and the pH was kept at 7.0 throughout the experiment. Nitrogen was used to flush the photobioreactors and ensure anaerobic conditions before each experiment. The schematic of the experimental setup is shown in Fig. 1.

### 2.2 Bacteria culture and its growth media

For the experiment, a pre-grown mixed culture of PNSB was used as the inoculum. The mixed culture had previously been cultivated for 20 days in a FSW that was nitrogen deficient. In this study, the FSW, with a composition described in Table 1, served as the carbon source, and was supplemented with a nitrogen-deficient nutrient solution to enhance the formation of PNSB biofilm. The nutrient solution included KH_2_PO_4_ (3.03 g/L), NaHCO_3_ (4.29 g/L), ATCC trace minerals (10 mL/L), and vitamins (10 mL/L) since FSW lacks these components. The use of nitrogen-deficient media was strategic, as the author's previous research has shown that such conditions enhance the formation of PNSB biofilm [19]. The details of the composition of the trace elements and vitamin solution used in the experiment are available in previous studies [19].

**Table 1:** General characteristics of FSW prior to the addition of nutrients showing mean and standard deviation for each parameter based on two replicates.

Parameter	Value	Unit
pH	3.35 ± 0	–
Electrical conductivity	206.2 ± 1.1	µs/cm
Oxygen reduction potential	279.5 ± 0.1	mV
Total COD	4500 ± 339	mg/L
Total carbon	1965.4 ± 0.3	mg/L
Total organic carbon	1951.2 ± 3.9	mg/L
Inorganic carbon	14.2 ± 3.5	mg/L
Total nitrogen	17.3 ± 9.6	mg/L
Organic acids	1123 ± 53	mg/L
Nitrate	17.56 ± 0.4	mg/L
Phosphate	231.3 ± 2.8	mg/L
Sulfate	13.4 ± 0.1	mg/L
Chloride	6.4 ± 2.2	mg/L

### 2.3 Analytical methods

The growth of PNSB in suspension was monitored by measuring its absorbance at 420 nm. The absorbance was measured with a UV-3600 plus spectrophotometer (Shimadzu, Japan). The final concentration of PNSB biomass (volatile suspended solids - VSS) from both suspended and biofilm growths was determined using the established gravimetric methods as described by the American Public Health Association (APHA, 2012). For quantifying the attached biomass, a specific volume of distilled water was used to wash off all the biofilm from agricultural shade cloth of each reactor, and this volume was recorded accordingly. To ensure representative sampling for total suspended solids (TSS) and VSS measurements, the washed-off biofilm was thoroughly homogenized in the water, ensuring even distribution of biofilm fragments before taking samples. The volume of water used for washing was then multiplied by the measured TSS and VSS values to calculate the final biomass mass.

The pH, COD, total organic carbon (TOC), total carbon (TC), inorganic carbon (IC), total nitrogen (TN), and anions (chloride, phosphate, and sulfate) were measured every third day throughout the experiment. The pH and oxygen reduction potential (ORP) were measured with a multimeter (Orion Star, Thermo Scientific, USA) immediately upon opening the reactor. COD was determined using the USEPA Reactor Digestion Method 8000 (Hach, 2019) and a DRB200 (Hach spectrophotometer, USA). TOC, TC, IC and TN were measured on a TOC-L Analyzer equipped with TNM-L unit (Shimadzu, Japan), and anions were measured by ion chromatography (940 Professional IC Vario, Metrohm, Switzerland).

Samples of the effluent wastewater were centrifuged at 5000 rpm for 10 min in a centrifuge (Sorvall 16R, Thermo Scientific, Germany) and filtered through a 0.2 µm Nalgene syringe filter to obtain the supernatant for the analysis of COD, TOC, TC, IC, TN, and anions. COD of the supernatant was determined on the same day of sample collection while other parameters were determined later, and the samples were stored at 4 °C until analysis.

At the end of the experiment, the Crts, BChls, protein, and PHB of the suspended and biofilm growth were extracted and analyzed. The Crts were determined using acetone as a solvent, BChls were extracted using an acetone/methanol (7:2 v/v) solvent. Protein was extracted and quantified using an alkaline extraction and the Lowry protein assay, and PHB was extracted and quantified using sodium hypochlorite dispersion and UV spectrophotometry, respectively. The detailed procedures used for extracting and analyzing these components have reported in a previous study [4].

### 2.4 Statistical analysis

To ensure data accuracy, biological duplicates were used. Statistical analysis was performed using a completely randomized design and analysis of variance (ANOVA) with a significance level set at 5 %. Between group comparisons following a significant ANOVA test used the conservative Bonferroni method. All statistical analyses were performed using Statistix 10 software.

## 3 Results

### 3.1 Impact of light intensity on biomass production

**Fig. 2:**
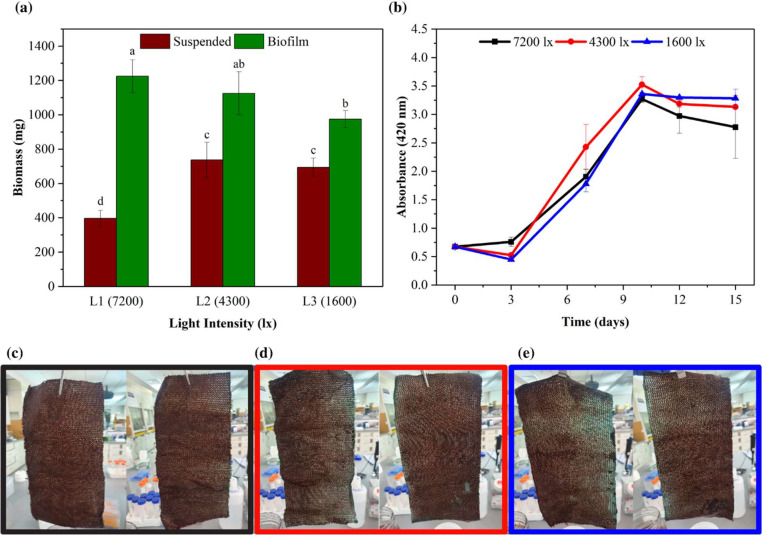
Comparison of biomass production, absorbance, and biofilm formation under three light intensities. (a) Biomass (VSS) from suspended and biofilm growth, (b) Absorbance profile, (c) Biofilm formation. Different alphabetical letters above columns represent significant differences from a Bonferroni post-hoc test conducted following a significant ANOVA test.

The results showed that the suspended growth of PNSB was greatest under 4300 lx conditions, with a biomass production of 736.7 ± 103.5 mg. The suspended biomass at 1600 lx was statistically similar (*p* = 0.668), at 694.2 ± 54.3 mg, while the suspended biomass under 7200 lx was significantly less at (396.7 ± 46.3 mg). For biofilm the 7200 lx condition produced the highest biomass production, with a value of 1225 ± 95.7 mg. Biofilm quantity decreased with decreasing light intensity, though the only statistically significant difference was between the 7200 lx and 1600 lx conditions (*p* < 0.006) (Fig. 2a), with the later producing 975 ± 50 mg of biofilm.

The biomass production from suspended and biofilm growth can also be measured via the absorbance value and biofilm images, respectively (Fig. 2b and c). However, absorbance is only indicative of PNSB growth, as biofilm cell detachment can occur changing suspended biomass concentrations and organisms can also alter their pigment concentrations depending on light availability and energy needs. On Day 3, the highest absorbance values were observed at a light intensity of 7200 lx (0.8 ± 0.08), indicating greater cellular metabolic activity and biomass production. This was followed by 4300 lx and 1600 lx. However, the absorbance value decreased over time under the 7200 lx intensity due to the development of biofilm on the agricultural shade cloth, which hindered light from reaching the cells in suspended growth. On Day 7, the highest absorbance was recorded at a light intensity of 4200 lx (2.4 ± 0.4), followed by 7200 lx and 1600 lx, and this trend persisted until Day 10. By Day 12, the highest values were recorded at a light intensity of 1600 lx (3.2 ± 0.00), followed by 4300 lx and 7200 lx (Fig. 2b), which remained constant until the end of the experiment.

### 3.2 Impact of light intensity on fuel-synthesis wastewater treatment

**Fig. 3:**
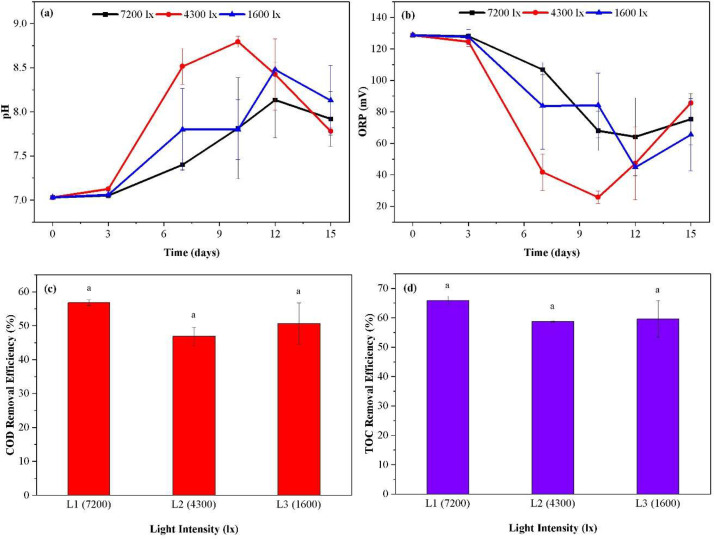
Comparison of pH and ORP profiles, and removal efficiency of pollutants (COD, and TOC) under three light intensities. (a) pH profile, (b) ORP profile, (c) COD removal efficiency, (d) TOC removal efficiency. Different alphabetical letters above columns represent a statistically significant difference from a Bonferroni post-hoc test following a significant ANOVA test.

The observed pH trends of the three experimental conditions exhibit a correlation with their corresponding absorbance values, and with the final total biomass produced. The 4300 lx showed the highest pH up until day 10 reaching 8.7 ± 0.06, whereafter it decreased steadily to reach 7.7 ± 0.04 at the end of the test. 1600 and 7200 lx followed a similar trend, increasing to between 8.4 and 8.1 and both peaking at day 12. Throughout, the 1600 lx was equal or greater than the 7200 lx (Fig. 3a). The oxygen reduction potential (ORP) displayed an opposite trend to that of the pH, signifying an inverse relationship between the two variables. The 4300 lx showed the lowest ORP until day 10 reaching 68 ± 12.5, whereafter it increased steadily to reach 75 ± 16.2 at the end of the test indicating readily biodegradable substrate had been consumed. 1600 and 7200 lx followed a similar trend, decreasing to between 128 and 84 and both bottoming at day 12. Throughout the test, the 1600 lx value was equal or lesser than the 7200 lx (Fig. 3b).

Fig. 3c–d shows the COD and TOC removal efficiencies of PNSB at three light intensities. The results obtained showed that the highest COD and TOC removal was observed at the highest light intensity, 7200 lx, with a COD removal of 56.8 ± 0.8 % and TOC removal of 65.8 ± 1.6 %. The lowest removals were recorded for the 4300 lx condition, with a COD removal efficiency of 46.9 ± 2.6 % and TOC removal of 58.8 ± 0.2 %. However, these differences between the conditions were not statistically significant. The efficacy of biomass production in relation to COD removal was assessed by calculating the VSS/COD ratio under different light intensity conditions. The analysis revealed that the VSS/COD ratio varied significantly with the intensity of light used. Specifically, the 7200 lx condition resulted in a VSS/COD ratio of 0.41 ± 0.0 g-VSS/g-COD, indicating the lowest efficiency of biomass in removing COD per unit of biomass produced. In contrast, the 4300 lx condition exhibited the highest efficiency, with a VSS/COD ratio of 0.58 ± 0.0 g-VSS/g-COD, suggesting that biomass produced under this light intensity was the most effective in COD removal. The 1600 lx condition yielded a VSS/COD ratio of 0.48 ± 0.1 g-VSS/g-COD, which, while lower than that at 4300 lx, was still higher than the efficiency observed at 7200 lx. However, there was no significant difference in the VSS/COD ratios among the different light intensity conditions.

### 3.3 Impact of light intensity on light conversion efficiency

Light is the energy source that drives the growth of PNSB, and the optimal light intensity should provide high light conversion efficiency to maximize productivity and minimize cost. The light conversion efficiency is calculated as the ratio of biomass production to light intensity used (Simionato et al., 2013), and is shown in Table 2. The values indicate that the maximum light conversion efficiency occurred at 1600 lx, although more biomass was produced at 7200 lx in biofilm growth and 4300 lx in both suspended and total growth.

**Table 2:** Light conversion efficiency comparison under three light intensities. Different alphabetical letters represent significant differences from a Bonferroni post-hoc test conducted following a significant ANOVA test.

Light Intensity (lx)	Light conversion efficiency (mg/lx)
L1 - 7200	0.23 ± 0.01 ^c^
L2 - 4300	0.43 ± 0.04 ^b^
L3 - 1600	1.04 ± 0.05 ^a^

### 3.4 Impact of light intensity on photopigments production

**Fig. 4:**
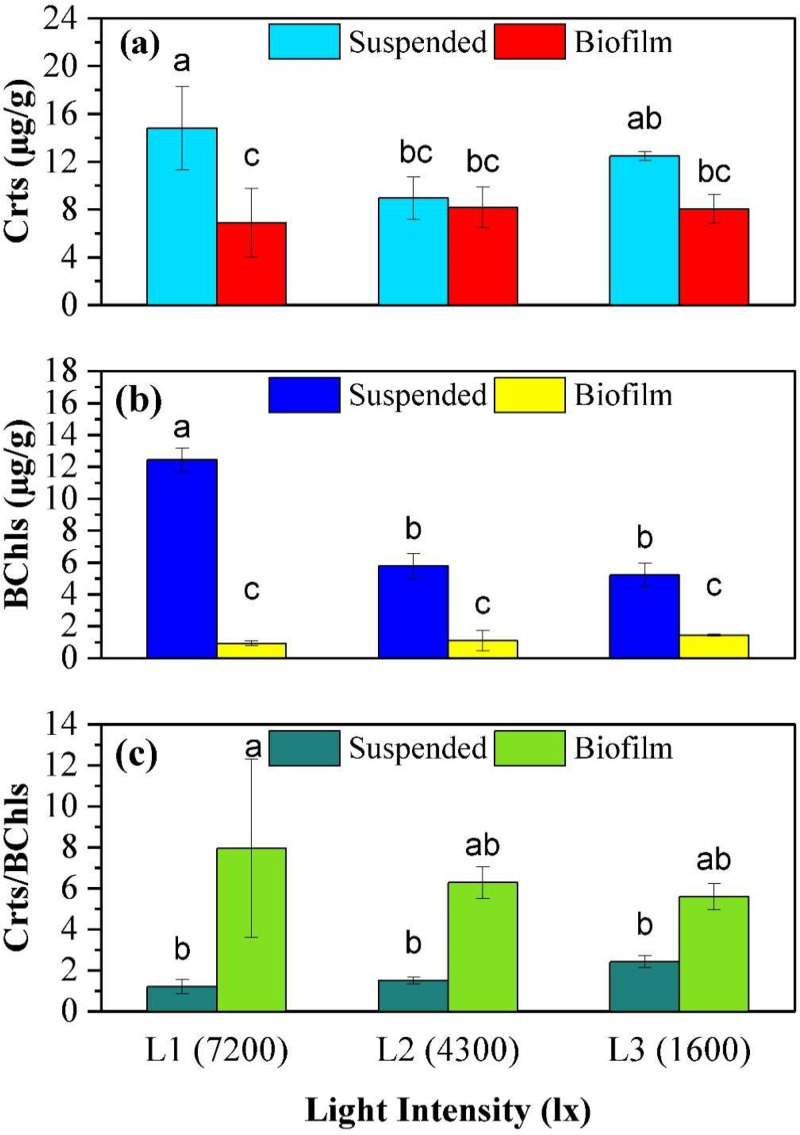
Photopigment measurements at the test completion from suspended and biofilm biomass. (a) Carotenoids, (b) Bacteriochlorophylls, (c) Carotenoids/Bacteriochlorophylls ratio. Different alphabetical letters above columns represent significant differences from a Bonferroni post-hoc test conducted following a significant ANOVA test.

Fig. 4 shows the trend in the concentration of Crts and BChls and their ratio as a function of light intensity and growth mode. In suspended growth, the Crts concentration was highest under 7200 lx light conditions with 14.8 ± 3.5 µg/g, while the lowest concentration was observed under 4300 lx with 8.9 ± 1.8 µg/g, showing inverse correlation to the suspended biomass quantity. In contrast, in biofilm growth, the Crts concentration was lowest under 7200 lx with 6.8 ± 2.8 µg/g, while it was slightly higher for the 4300 lx and 1600 lx conditions with 8.2 ± 1.7 µg/g and 8.0 ± 1.2 µg/g, respectively. The only statistically significant differences were for the Crts concentration of the suspended growth between the 7200 lx and 4300 lx conditions (*p* = 0.01).

The BChls concentration for suspended biomass was highest under 7200 lx with 12.4 ± 0.7 µg/g and lowest under 4300 lx and 1600 lx with 5.7 ± 0.8 µg/g and 5.2 ± 0.7 µg/g, respectively. The 7200 lx was found to be significantly different (*p* < 0.001) from both 4300 lx and 1600 lx conditions. The BChls concentration were much lower in the biofilm, with the minimum of 0.9 ± 0.2 µg/g occurring under the 7200 lx condition. The 4300 lx condition was similar at 1.0 ± 0.6 µg/g while the 1600 lx condition reached 1.4 ± 0.1 µg/g. However, for biofilm growth, no significant difference (*p* > 0.80) existed between all three light conditions.

The Crts/BChls ratio varied depending on the light conditions and growth mode of the organisms. The highest Crts/BChls ratio was obtained from suspended growth under 1600 lx conditions (2.4 ± 0.3). For biofilm growth, the Crts/BChls ratio was found to be relatively high compared to suspended growth under all three light intensity conditions (7.9 ± 4.4, 6.3 ± 0.7, and 5.6 ± 0.6, respectively).

### 3.5 Impact of light intensity on protein production

**Fig. 5:**
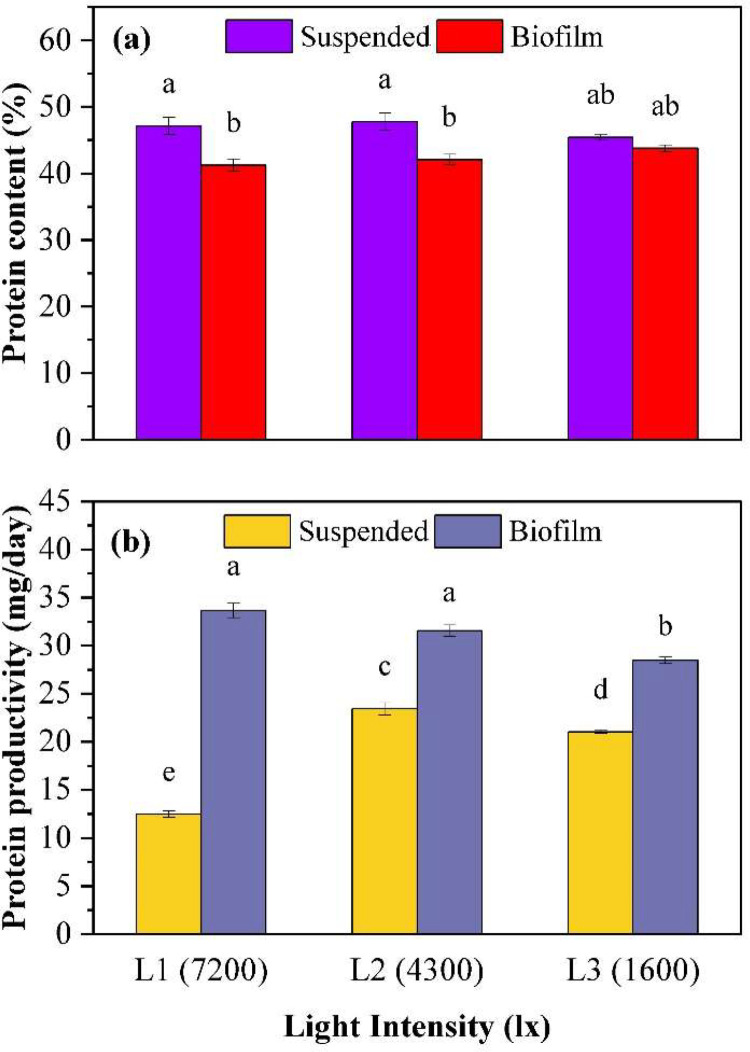
Comparison of protein content and productivity under three light intensities. (a) Protein content, (b) Protein productivity. Different alphabetical letters above columns represent significant differences from a Bonferroni post-hoc test conducted following a significant ANOVA test.

The cellular protein percentage obtained from the PNSB in suspended and biofilm growth under different light intensity conditions was analyzed in the experiment. The results showed that there was no significant difference (*p* > 0.25) in the cellular protein percentage obtained from suspended growth under all three light intensity conditions (7200, 4300, and 1600 lx). Similarly, there was no significant difference (*p* > 0.19) in the cellular protein percentage obtained from biofilm growth under all three light intensity conditions. In suspended growth, the protein percentages ranged from 45.4 % to 47.7 %, while in biofilm growth they ranged from 41.2 % to 43.7 % (Fig. 5a)

The SCP productivity in suspended growth was significantly different (*p* < 0.02) between the three light intensity conditions (7200, 4300, and 1600 lx). The highest SCP productivity was observed in the 4300 lx condition with a value of 23.4 ± 0.6 mg/d, followed by 1600 lx with 21.0 ± 0.2 mg/d and 7200 lx with 12.5 ± 0.3 mg/d. In biofilm growth, the SCP productivity was also affected by light intensity. The highest SCP productivity was observed in the 7200 lx condition with a value of 33.6 ± 0.7 mg/d, followed by 4300 lx with 31.5 ± 0.6 mg/d, and 1600 lx with 28.5 ± 0.3 mg/d. The 1600 lx condition was significantly different (*p* < 0.007) to the other two lighting conditions (Fig. 5b).

Table 3 shows the total (suspended + biofilm) SCP production. The results indicated that the total SCP production varied between the three light intensity conditions. The 4300 lx condition showed the highest total SCP production with a value of 825 ± 0.8 mg, which was significantly different from the total SCP production from the 7200 lx and 1600 lx conditions (*p* < 0.03). The 7200 lx condition produced a total SCP of 692 ± 16 mg, while the 1600 lx condition produced 741 ± 7.4 mg. No significant difference was observed between the total SCP production of the 7200 lx and 1600 lx conditions.

**Table 3:** Total SCP and PHB production comparison under three light intensities. Different alphabetical letters represent significant differences from a Bonferroni post-hoc test conducted following a significant ANOVA test.

Light intensity (lx)	Total SCP production (mg)	Total PHB production (mg)
L1 - 7200	692 ± 16 ^b^	191 ± 6 ^a^
L2 - 4300	825 ± 0.8 ^a^	180 ± 12 ^a^
L3 - 1600	742 ± 7.4 ^b^	198 ± 25 ^a^

### 3.6 Impact of light intensity on PHB production

**Fig. 6:**
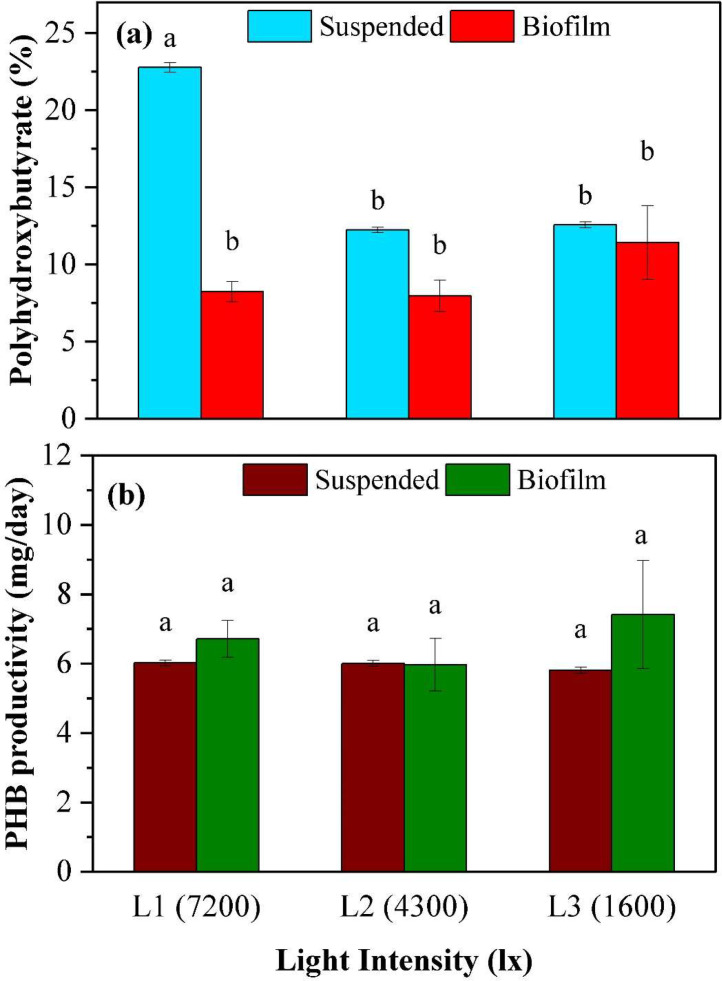
Comparison of PHB content and productivity under three light intensities. (a) PHB content, (b) PHB productivity. Alphabetical letters represent significant differences from a Bonferroni post-hoc test conducted following a significant ANOVA test.

In the suspended growth, the highest light intensity (7200 lx) resulted in the highest PHB content (22.7 ± 0.3 %), significantly different (*p* < 0.001) from other two light intensities (4300 lx: 12.2 ± 0.2 % and 1600 lx: 12.6 ± 0.2 %). In contrast, the biofilm showed no significant difference (*p* > 0.12) in PHB content among the three light intensities. The highest light intensity (7200 lx) resulted in PHB content of 8.2 ± 0.7 %, while the 4300 and 1600 lx conditions produced PHB contents of 7.9 ± 1.0 % and 11.4 ± 2.4 %, respectively (Fig. 6a).

The results of the PHB productivity in suspended and biofilm growth showed that the productivity remained similar (*p* > 0.45) across the tested light intensities. In suspended growth, the productivity ranged from 5.8 to 6.0 mg/d, while in biofilm growth, it ranged from 5.9 to 7.4 mg/d (Fig. 6b). The total PHB production was also found to be similar (*p* > 0.5) for the three light intensities, ranging from 180 to 198 mg for the three light conditions (Table 3).

## 4 Discussion

The purpose of this study was to investigate the impact of light intensity on the treatment of FSW using PNSB in a hybrid biofilm-suspended growth system, and the subsequent recovery of value-added bioproducts.

### 4.1 Impact of light intensity on biomass production and wastewater treatment

Our findings indicate that light intensity significantly influences both the biomass production of PNSB and the treatment of FSW. Suspended growth was greatest in 4300 lx and 1600 lx conditions, while biofilm growth was maximal under 7200 lx conditions. The greater suspended growth at 4300 lx and 1600 lx appears to be due to the reduced formation of biofilm on the agricultural shade cloth under these conditions, which allows more light to penetrate and promote higher levels of suspended growth. Conversely, the greater biofilm biomass production at 7200 lx seems to stem from the increased availability of light energy, which enhances metabolic processes and supports a thicker biofilm growth, blocking light passage into the suspended growth culture. This is supported by the observation that low levels of suspended growth biomass production were seen at 7200 lx. This was also seen in the transition of the 7200 lx reactor, which at Day 3 had achieved the highest absorbance values. As biofilm developed, shading prevented the suspended biomass from growing, resulting in the subsequent decline in absorbance.

These findings suggest a complex interplay between light intensity, biofilm formation, and suspended growth. While higher light intensities provide greater light penetration that can promote biofilm formation and growth, they can also lead to shading effects that reduce light availability for suspended cells. It is important to note in this study the biofilm support material was closest to the light source, and different growth trends between suspended biomass and biofilm may be observed if sufficient space is provided for suspended growth between the light source and biofilm support material.

Interestingly, the highest biomass production at 4300 lx resulted in the lowest organic removal. This can most likely be attributed to the pH fluctuations observed in this condition, which spent a considerable period over 8.5, while the other two conditions did not reach 8.5 throughout the test. The elevated pH values could have impacted the bacteria's metabolic processes, inhibiting bacterial activity and thereby reducing COD and TOC removal.

In terms of wastewater treatment, the highest COD and TOC removal efficiencies were observed at the highest light intensity, 7200 lx. This is consistent with previous studies [20,21,17] that have shown that light intensity can affect the photosynthetic process in phototrophic bacteria, leading to an increase in the production of cellular components and ultimately the removal of COD and TOC from the wastewater. However, it is important to note that there is a threshold limit beyond which the light intensity has no further effect on the removal efficiency [22]. While the 7200 lx condition achieved the highest overall COD removal, the VSS/COD ratio shows that the biomass produced under this condition was less efficient in removing COD per unit of biomass compared to the lower light intensities. The highest biomass yield (VSS/COD ratio) was observed at 4300 lx. This highlights that while higher light intensities may enhance overall removal efficiencies, they do not necessarily optimize the efficiency of biomass production. This efficiency trade-off suggests that intermediate light intensities, such as 4300 lx, might provide a better balance between biomass production and effective COD removal, avoiding the diminishing returns seen at the highest light intensities. Although the biomass growth relative to light input was highest at the lowest light intensity (1600 lx), the biomass produced under this condition was less efficient in removing COD, as indicated by a moderate VSS/COD ratio. Conversely, the 4300 lx condition had a moderate growth relative to light intensity but, coupled with its high biomass production and substrate yield, offers the most effective balance between biomass production, COD removal efficiency and light inputs.

These findings suggest that light intensity plays a crucial role in the treatment of FSW using PNSB. However, the optimal light intensity may depend on various factors, including the specific wastewater being treated, the bacteria strain used, and other environmental factors. Therefore, it is necessary to consider the interactions between light intensity and these other factors to optimize the treatment of FSW using PNSB.

### 4.2 Impact of light intensity on bioproduct recovery

#### 4.2.1 Photopigments production

In photosynthetic microorganisms, light harvesting complexes or photosynthetic pigments are responsible for absorbing most of the light energy. Crts and BChls are the dominant pigments [23] that aid in the capture and transformation of light into chemical energy in PNSB. Both pigments exist within cells and are associated with the protein components of the photosynthetic system on the cell membrane [24]. During periods of excessive light, Crts play a major role in protecting the cells by absorbing excessive light energy. The protective abilities of Crts vary based on their specific chemical and structural properties [25].

BChls showed a clear increasing trend with increasing light intensity in the suspended growth, but remained relatively unchanged in biofilm growth. Considering the shading effects of the biofilm, which was greatest in the 7200 lx and least in the 1600 lx condition, this can explain the greater BChl production at higher light intensities. This enables the suspended growth to capture sufficient energy from reduced light available behind the biofilm where the suspended growth primarily occurs.

The relationship for Crts was less clear. The complex behavior can be understood by considering that Crts can act both in a photoprotection role under excessive light and a photo-harvesting role in limited light [26]. The photoprotection role is further complicated by two possible situations suspended biomass may face in the reactor. Most of its time is spent in suspension behind the biofilm, but due to a small gap between the biofilm and vessel wall, suspended cells can be exposed to significantly higher light levels for brief periods of time. These complex range of factors may drive the variation in Crts seen between the reactors. The highest Crts were observed in the 7200 lx high light intensity reactor and are therefore likely associated with a response to the short-term photoprotection. At lower light intensities, and higher suspended biomass concentrations photoprotection may be more driven by long-term or average conditions experienced behind the biofilm.

The limited changes seen in both Crts and BChls in biofilm may be associated with the cross-sectional average. It is expected that cells near the surface would be expressing high in Crts for photoprotection due to their constant proximity to the light source, while cells deeper in a biofilm would be expressing higher in BChls to aid in light harvesting of any photons that manage to penetrate. Since it was observed that the biofilm is thicker at high light intensity there would be a greater requirement for Crts at the surface and less requirement for BChls. However, the thicker biofilm would create the reverse situation at the biofilm-support media interface, leading to an overall averaging effect.

The ratio of Crts to BChls primarily differed based on the growth mode, with a higher ratio prevalent in the biofilm. This is linked to the closer proximity to the light source, and therefore greater need for photoprotection and reduced need to increase photoharvesting. The discrepancy between the biofilm and suspended growth reduced as the light intensity reduced. Under reducing light intensity, the biofilm thickness decreased and therefore differences in light conditions between the outer and inner reactor also reduced, leading to more similar ratios.

This study's results align with our prior study [4], where higher levels of Crts and BChls were obtained in suspended growth and a higher Crts/BChls ratio was found in biofilm growth. However, the concentration of Crts and BChls in this study was higher than in our previous study, which could be due to the higher light intensity (12,500 lx) used previously. In contrast to this study, Hülsen et al. [6] found higher concentrations of both pigments in biofilm growth rather than suspended growth, which was attributed to biofilm growth on the light source, the use of IR light which is quickly attenuated in liquid, and different light intensity.

#### 4.2.2 Protein production

Cellular protein content remained relatively consistent across the three light intensities for both growth modes. This suggests that the cellular protein synthesis mechanisms in PNSB are robust, with light intensity having minimal influence on protein content. However, a slight difference in protein percentages between suspended (45.4 % to 47.7 %) and biofilm growths (41.2 % to 43.7 %) was observed. This could be related to reduced growth rates in biofilms [27] or due to increased extracellular polymeric substances within the biofilm that may have reduced protein content. Given the small differences in protein content, SCP productivity is primarily driven by the response of biomass growth to light.

#### 4.2.3 PHB production

The influence of light intensity on PHB content in PNSB's suspended growth is evident, with the 7200 lx condition yielding the highest PHB content. This marked difference from the other two light intensities underscores the potential of higher light intensities to enhance PHB production in suspended growth. As photosynthesis transforms light energy into chemical energy, it generates the essential reducing equivalents and ATP. When these are in excess, PHB synthesis is one route for spilling these excess equivalents and maintaining cell homeostasis [28]. The pronounced PHB production under 7200 lx is further amplified by the minimal suspended biomass concentration, which increases the photon energy available per cell.

For biofilm the PHB content remained relatively unaffected by the varying light intensities. Even at the highest light intensity, 7200 lx, the PHB content was comparable to the other conditions. A plausible explanation relates to the thickness of the biofilm and the proportion of biomass that receives excess light to drive PHB synthesis. As the light intensity and biofilm thickness increase, cells at the surface of the biofilm may produce higher quantities of PHB, but those deeper have insufficient reducing equivalents, leading to a similar overall PHB yield.

Regarding PHB productivity, both suspended and biofilm growths exhibited comparable rates across the tested light intensities. This suggests that the most effective light intensity for PHB productivity might not necessarily be the highest. Furthermore, the overall PHB production remained consistent across the three light intensities, hinting at a potential equilibrium between cell PHB content and biomass productivity. This is further confirmed by the strong negative correlation between these two variables, with a Pearson correlation coefficient of -0.88. Future study is recommended to evaluate whether the costs involved in supply of additional lighting, either through artificial sources or by increasing the photobioreactor surface area can be offset by reduced PHB harvesting costs associated with a reduced total biomass and increased cellular PHB content.

### 4.3 Economics of recovery

The economic viability of recovering value-added bioproducts from FSW treatment using purple phototrophic bacteria under different light intensities in a hybrid biofilm-suspended growth system is influenced by the light intensity used in the process.

Our study revealed that no significant differences were observed in the highest COD removal efficiency, Crts, protein, PHB (biofilm), and BChls (biofilm) production. This suggests that, for the economic feasibility of the process, a light intensity of 1600 lx is the most favorable condition due to either its lower energy use for a given PBR design, or wider/deeper PBR for the same available light. However, costs of extraction, particularly with PHB, must also be considered, which become more cost-effective as cellular concentrations increase.

To further improve the economic feasibility of this process, utilizing sunlight as a light source for PNSB growth can significantly reduce operating costs by eliminating the need for artificial light sources. The average light intensity reaching the surface of earth in the study location of Qatar varies from 650 W/m^2^ in winter and 1000 W/m^2^ in summer (Sajid and Bicer, 2023), which is much higher than the maximum light intensity tested in this study 7200 lx (56 W/m^2^). Therefore, to optimally utilize sunlight as light source, use of deep reactors, sun-tracking systems and shading devices may be required to optimize the light intensity.

Further complications from natural lighting include its variation in light intensity and spectra with time, and associated temperature changes. To address these uncertainties, it will be important to carefully monitor light intensity, spectra and temperature in long-term outdoor experiments to enable data mining of how these parameters influence bioproduct production.

## 5 Conclusions

In conclusion, this study sheds light on the influence of light intensity on the treatment of FSW and the recovery of valuable bioproducts from PNSB enriched suspended and biofilm biomass. Based on the results, it appears that a light intensity of 1600 lx may offer a more economical approach for producing certain bioproducts, despite a slight reduction in production rates compared to higher light intensities of 7200 lx and 4300 lx. However, the results indicate that a high light intensity of 7200 lx leads to improved biofilm production, as well as the highest concentrations of Crts, BChls, and PHB in suspended biomass. Importantly, the study highlights that cellular protein content is not affected by light intensity directly, though its effects are noticed indirectly in the form of growth (biofilm or suspended) that it promotes and their relative growth rates. Hence, the findings reveal that different light intensities have varying impacts on the treatment process and the production of bioproducts. The results highlight that the optimal light intensity for FSW treatment and bioproduct recovery depends on the specific goals of the process, and that careful consideration of light intensity is crucial in achieving maximum results. The light intensity impact on FSW treatment and bioproduct recovery is a complex interplay between various factors, including initial light intensity, PNSB growth, and shading effects between both biofilm and suspended growth that may vary depending on reactor layout. This study contributes to a deeper understanding of these relationships and provides insights into the design and optimization of photobioreactors for sustainable bioproduct production from FSW.

## Funding

This work was supported by 10.13039/100008982Qatar National Research Fund through the National Priorities Research Program (Grant number: NPRP11-S-0110-180245). Open access funding provided by the Qatar National Library.

## CRediT authorship contribution statement

**Sultan Shaikh:** Writing – review & editing, Writing – original draft, Visualization, Validation, Methodology, Investigation, Formal analysis, Data curation, Conceptualization. **Gordon McKay:** Writing – review & editing, Supervision. **Hamish Robert Mackey:** Writing – review & editing, Supervision, Resources, Project administration, Funding acquisition.

## Declaration of competing interest

The authors declare that they have no known competing financial interests or personal relationships that could have appeared to influence the work reported in this paper.
